# Movement Disorders and Dementia in a Woman With Chronic Aluminium Toxicity: Video-MRI Imaging

**DOI:** 10.5334/tohm.588

**Published:** 2021-02-01

**Authors:** Antonio Jose Reyes, Kanterpersad Ramcharan, Stanley Lawrence Giddings, Amrit Ramesar, Edmundo Rivero Arias, Fidel Rampersad

**Affiliations:** 1 Neurology Unit; Department of Medicine, San Fernando Teaching Hospital, The University of the West Indies, TT; 2 Department of Medicine, Surgi-Med Clinic, San Fernando, TT; 3 Department of Medicine, San Fernando Teaching Hospital, The University of the West Indies, TT; 4 Radiology Department, The University of the West Indies, St. Augustine, TT

**Keywords:** Encephalopathy, movement disorders, dementia, aluminium, paints

## Abstract

**Background:**

Aluminium encephalopathy results from exposure to aluminium from occupational, recreational, and environmental sources. Movement disorders, cerebellar ataxia, pyramidal tract signs, dementia, microcytic anemia and bone disease are typical manifestations.

**Case Report:**

A 55-year-old woman had clinical manifestations, persistent hyperaluminemia without magnetic resonance imaging (MRI) scan changes of toxic encephalopathy following a prolonged exposure to marine grade paints containing 30% aluminium. Chelation therapy with ethylenediaminetetraacetic acid (EDTA) demonstrated decreased levels of aluminemia and significant neurological improvement over time.

**Discussion:**

This diagnosis should be entertained in patients with movement disorders, cerebellar ataxia, pyramidal signs, and dementia of unknown etiology.

**Highlights:**

Aluminium encephalopathy (AE) is a neurological syndrome caused by aluminium neurotoxicity. Manifestations include cognitive impairment, motor dysfunction, microcytic anemia and bone disease. This case illustrates AE with hyperaluminemia associated with chronic exposure to industrial paints and clinical and biochemical reversibility after chelation therapy with ethylenediaminetetraacetic acid. Movement disorders are highlighted.

## INTRODUCTION

The neurological syndrome associated with chronic toxicity by aluminium in adults has been most investigated in patients receiving regular hemodialysis, bladder irrigation with 1% alum, intramuscular injections of vaccines containing aluminium, among workers of the aluminium potroom industry, and drug addicts exposed to intravenous injections of a methadone solution “cooked” in an aluminium pot [1,2,3,4,5,6,7,8,9,10]. Recognized neurological features are dementia (98%), speech disturbances (95%), myoclonus (81%), epileptic seizures (57%), and psychotic episodes (52%) [6]. Intention tremor, dysmetria, dysdiadochokinesia and ataxia have also been reported [6,7,8,9,10]. We report a 55-year-old woman, who presented with these abnormal movement disorders, cerebellar ataxia, pyramidal signs, and dementia consistent with an encephalopathy caused by aluminum toxicity and highlight bilateral blepharospasm, polyminimyoclonus and dystonic posturing of the hand as novel features [1,2,3,4,5,6,7,8,9,10,11,12,13,14,15,16,17,18,19,20,21,22,23,24,25].

## CASE REPORT

A 55-year-old woman with a 12-year history of exposure to marine grade paints containing 30% aluminium presented to the emergency room in 2014 with a history of progressively worsening dementia, cerebellar ataxia, and abnormal movement disorders. These involuntary movement disorders were characterized by polyminimyoclonus with dystonic posturing of the left hand, intention tremor of the right hand, tremor of the head and bilateral blepharospasm, dysdiadochokinesia and impaired finger tapping bilaterally, and dysmetria with spastic ataxic gait and ample sustentation base (***Video 1 segment 1, 2***). Several distractibility maneuvers were performed on the patient such as multitasks interference of the movements using abrupt auditory and tactile stimuli in various regions of the body and presentation of different images at the same time. There was no change in the amplitude, duration, cessation or increase of the abnormal movements observed or in their patterns. Lack of entrainment of the tremor was also noted using the clinical method in which the clinician sits opposite the patient and perform a tremor examination looking for tremor at rest, on posture and during action. The patient was placed in the position in which there were the most tremors, with the arms out in front. The least affected limb (left upper limb) was moved slightly away from the other and the patient was asked to focus on it. The patient was asked to tap with her left thumb and forefinger at different rhythmical frequencies (faster and slower) set by the clinician. We assessed the changes in the amplitude, duration, cessation or increase of the tremor of the right hand.

**Video 1:** **Movement disorders phenomenology:** In sequence, **Video 1 segment 1 pre-treatment condition recorded in 2014** shows polyminimyoclonus with dystonic posturing of the left hand, tremor of the right hand, tremor of the head and bilateral blepharospasm, dysdiadochokinesia and impaired finger tapping bilaterally. The tremor usually commences in the first hour of the wake and remains unchanged throughout wakefulness. This involuntary motor activity consisted of abnormal, slow, segmental, continuous, multifocal and predominantly involving the distal muscles. There is an observable pattern of 1 to 2 sequences of muscle contractions every 2 to 3 seconds continuously during day or night. The tremor stops when patient grasps an object or a body part or when she places the hands firmly on a surface. **Video 1 segment 2 pre-treatment condition recorded in 2014** exhibits dysmetria and ataxic gait (Ambulation assisted using a cane). **Video 1 segment 3 post-treatment condition recorded from 2015 to 2020** demonstrates movements of the upper limbs and hands markedly diminished in amplitude and intensity bilaterally, no detectable head tremor or blepharospasm, marked improvement in dysdiadochokinesia, finger tapping bilaterally and dysmetria, and ambulation without assistance.

There was an insidious progression of the syndrome over ten years after an acute occupational exposure to paint vapors in 2004, which led to an acute chemical pneumonitis that required mechanical ventilatory support for 5 days. Investigations in 2004 revealed a very high level of serum aluminium at 1,320 µg/L (reference values 0 to 9 µg/L). The patient recovered from that event and resumed working as a cleaner in the same yacht paint company after 2 weeks of sick leave. No further medical interventions or warning was given to the patient despite her hyperaluminemia. She was unaware on the accumulative chronic exposure to neurotoxins or the need to use personal protective equipment to reduce exposure to paint vapors. Neither skin nor respiratory protection was provided. Notification of risks and a workplace chemical risk assessment were not conducted. The patient had no history of previous trauma, medical, surgical or neurological condition and had no history of use of recreational drugs, alcohol, or medication. Further history demonstrated no evidence of seizures, incontinence, orthostatic hypotension, visual or hearing loss. There was no family history of neurological diseases. On admission in 2014, the patient scored 5/30 on the Mini Mental State Examination (MMSE) test with significant impairment in abstract, reasoning, drawing, and writing. The written tasks associated with the MMSE test were also impaired by the involuntary movement disorders of both hands. She was alert, oriented to time person and place with a Glasgow coma scale of 15/15. She had normal vital signs with a body mass index (BMI) of 30 kg/m^2^. Ophthalmological examination was normal. There was no nystagmus or dysarthria. Muscle power was 3/5 in all limbs using the Medical Research Council scale. There was hyperreflexia in both lower limbs, and proprioception and vibration loss in upper limbs. The Romberg’s sign was positive. There was no clinical evidence of parkinsonism. The rest of the physical examination was unremarkable. As her condition was chronic, she was discharged for outpatient investigations. She was declared disabled for further work in 2014.

Extensive medical investigations and follow up were conducted for 6 years since 2014 ruling out most of the differential diagnoses (table 1, 2 submitted as supplementary files 1, 2).

**Figure 1:**
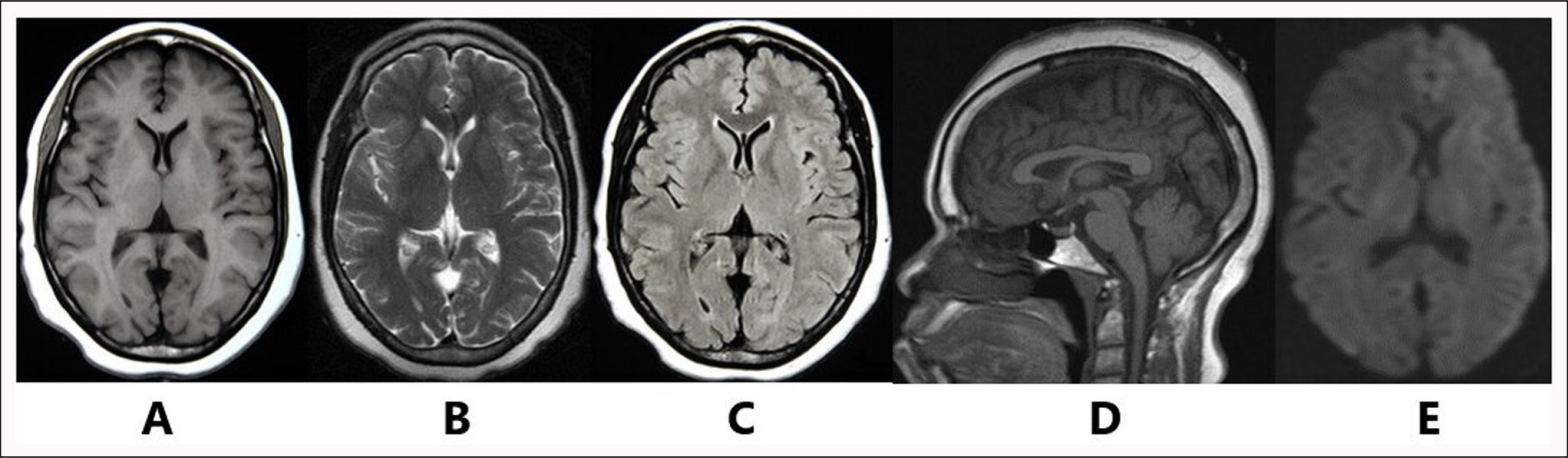
Brain MRI shows non-enhancing bilateral increased signal intensity on axial T1-weighted **(A)**, axial T2-weighted **(B)** and axial T2-Fluid-attenuated inversion recovery (FLAIR)-weighted **(C)** images in periventricular regions, basal ganglia and internal/external capsules. Mild cerebral and cerebellar atrophy is exhibited in (A), (C), and (D). Sagittal T2-weighted image **(D)** shows normal corpus callosum, brainstem, and cervical-medullary junction. Axial diffusion-weighted image demonstrates no areas of restricted diffusion to suggest ischemia/infarction **(E)**.

**Figure 2:**
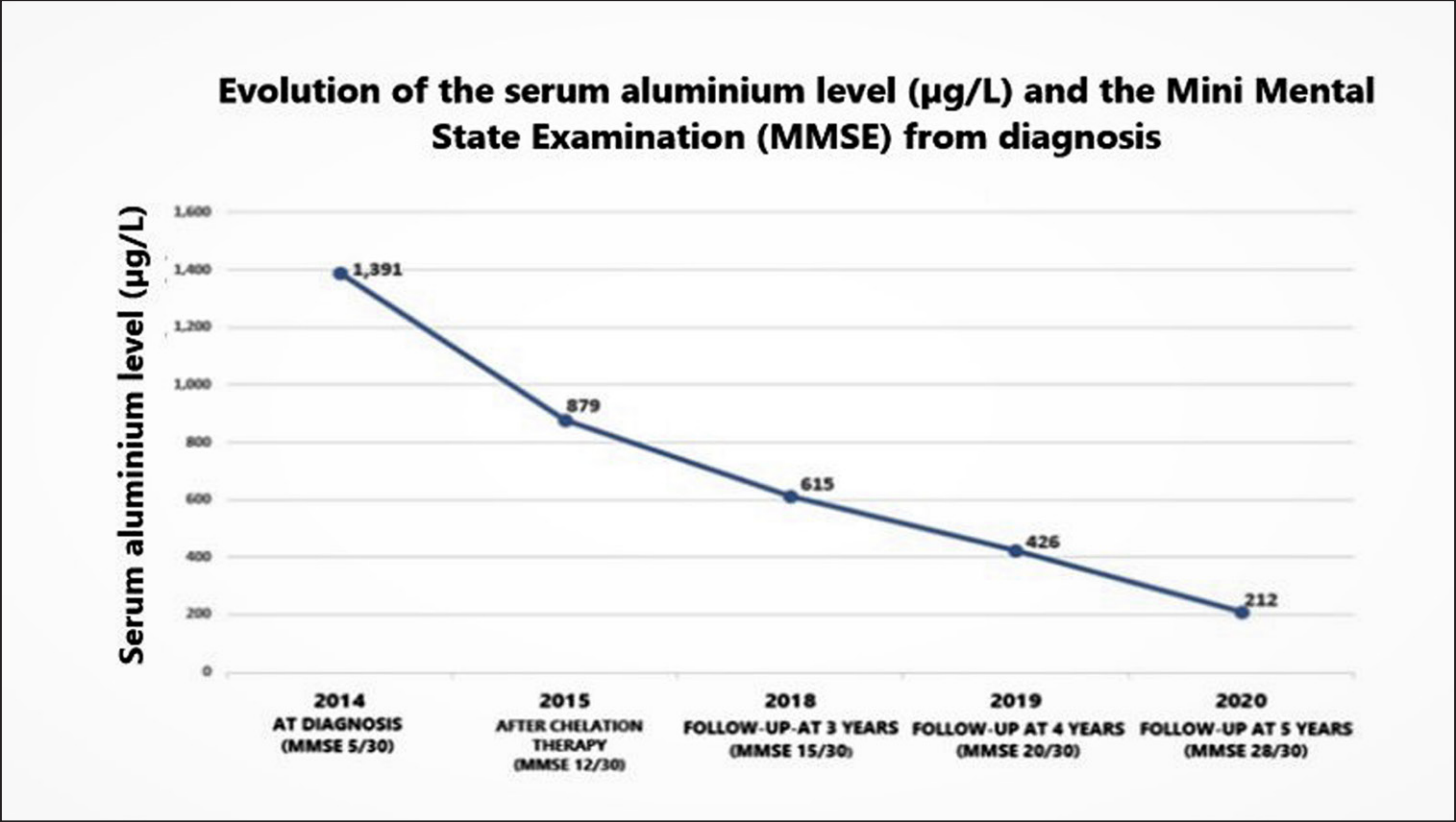
Schematic diagram of the timeline of events in relation to serum aluminium level and Mini Mental Status Examination (MMSE).

Blood tests revealed microcytic anemia with normal iron studies, vitamin B12 and folate blood levels. Cerebrospinal fluid (CSF) analysis was normal. The polymerase chain reaction (PCR) test of the CSF for multiple infectious diseases was negative (table 1 submitted as supplementary file 1). A scalp electroencephalogram (EEG) and an electromyography/nerve conduction studies were normal. Additional formal neuropsychological testing was unavailable. MRI scan in 2014 showed likely mild cerebral and cerebellar atrophy, with sparing of the corpus callosum, brainstem and cervical-medullary junction. There was non-enhancing bilateral increased signal intensity on T1-weighted, T2-weighted or T2-weighted Fluid-attenuated inversion recovery (FLAIR) images in the periventricular regions, basal ganglia and internal/external capsules to suggest chemical toxicity, and no areas of restricted diffusion were identified to suggest ischemia/infarction (***Figure 1***). The cervical, thoracic, and lumbosacral spine MRI scans were normal. Brain magnetic angiography (MRA) was also normal. Based on these neurological manifestations and natural history, movement disorder phenomenology, extensive investigations ruling out differentials, persistent hyperaluminemia, and workplace chemical exposure analysis, the patient was diagnosed with encephalopathy and movement disorders caused by the chronic exposure to multiple mixed paints containing high grade aluminium. On admission in 2014, the serum level of aluminium was high at 1,391 µg/L with a MMSE score of 5/30. The patient had chelation therapy with 1 gram of EDTA powder given sublingual twice daily for 30 days. This therapy was repeated 3 times between the years 2014 and 2015 using the protocol previously described. The patient demonstrated a gradual improvement of the motor dysfunction and decreased aluminemia to 879 µg/L with improvement in the MMSE score to 12/30 after treatment. Progressive improvement of her cognition, memory, and motor dysfunction throughout the year 2015 to 2019 was documented. At three and four years follow up, we noted an exponential fall of aluminemia at 615 and 426 µg/L and MMSE scores improved to 15/30 and 20/30 respectively. By the year 2020, a MMSE test scored 28/30, serum aluminium level dropped to 212 µg/L accompanied by complete resolution of tremor of the hands, polyminimyoclonus and dystonic posturing of the left hand, tremor of the head, and blepharospasm, with significant improvement in finger tapping impairment, dysdiadochokinesia, dysmetria, ataxic gait and motor weakness (***Video 1 segment 3***), without subsequent relapse up to the time of writing. ***Figure 2*** summarizes the timeline of events in relation to serum aluminium levels and the MMSE scores plotted against time. The patient has not received any therapy other than chelation with EDTA, to date of writing.

## DISCUSSION

Aluminium encephalopathy results from the interaction of aluminium with the CNS to produce inflammation, degeneration, and demyelination [1,2,3,4,5,6,7,8,9,10,11]. Although many neurotoxins such as lead, mercury, aluminum, copper, zinc, recreational drugs, alcohol, and tobacco are present in our environment, only a few of them cause pathognomonic neurologic syndromes [1,2,3,4,5,6,7,8,9,10,11,12,13,14,15,16,17,18,19]. Most of their manifestations can mimic psychiatric, metabolic, inflammatory, neoplastic and degenerative diseases of the nervous system (table 2 submitted as supplementary file 2) [1,2,3,4,5,6,7,8,9,10,11,12,13,14,15,16,17,18,19]. So, awareness of AE among clinicians is essential for early diagnosis.

Differentials of AE include Creutzfeldt-Jakob disease (CJD), multiple system atrophy (MSA), CNS infections, autoimmune encephalitis, metabolic encephalopathy and neurodegenerative syndromes [1,2,3,4,5,6,7,8,9,10,11,12,13,14,15,16,17,18,19]. A significant and permanent partial neurological and biochemical reversibility after chelation with EDTA is against the diagnosis of CJD, MSA, and other degenerative neurological syndromes. Clinical follow up, CT and MRI scans findings and other investigations pre-therapy and an improved MMSE test score of 28/30 post-therapy ruled out those diseases [1,2,3,4,5,6,7,8,9]. The EEG characteristic pattern in AE among patients on long term hemodialysis is also present in encephalopathy or neurodegeneration, and it is characterized by a slow background with superimposed bursts of high-amplitude slow waves, sharp waves, and complexes of spikes and slow waves [14]. However, the EEG can also be normal in AE especially during the reversible therapeutic window such as in our patient [1,2,3,4,5,6,7,8,9, 14].

To date, our patient has not exhibited autonomic involvement or parkinsonism. The CT scans of the chest, abdomen, and pelvic, and the CNS MRI scan findings ruled out space occupying lesions, stroke, and neoplastic diseases. Extensive blood and radiological investigations ruled out infectious, immunological, endocrine, nutritional, vasculitis, and hematological diseases. CSF real time PCR tests ruled out CNS infections. The neurological manifestations, medical investigations and MRI scan imaging features were not consistent with a paraneoplastic or toxic myelopathy [13]. Heavy metals such as lead, mercury, arsenic, and chromium levels in blood were normal. Despite cerebellar dysfunction, there were no peripheral polyneuropathy, hearing or visual deficits, visual fields defects or optic neuropathy and no history of exposure to manganese, methanol, carbon disulfide, cyanide, organic tins or methyl bromide [13].

Aluminium is the most abundant metal on our planet and the risk of exposure is occupational and environmental, especially during the refining of bauxite, production of alumina and aluminum and other secondary industries where the metal is used such as in the transportation, building and construction, packaging, electrical equipment, pharmaceuticals, food additives, cosmetics and other household products industries [1,3,7,8,9].

The mechanism by which the skeleton binds aluminium is unknown. The aluminium ion (Al3+) binding occurs only in the actual mineralization zone of the osteoid. The increase of aluminium found in the spinal cord and brain have been reported among patients with Alzheimer’s disease (AD), multiple sclerosis (MS), amyotrophic lateral sclerosis (ALS), and other neurodegenerative diseases [1,2,3,4,5,6,7,8,9,10,11,12,13,14,15,16,17,18,19]. The late onset and insidious deterioration of brain function are also consistent with a slow poisoning by aluminium ion (Al3+) and accumulation [1,3,7,8,9]. AE can be potentially reversible with therapy or removal of the offending agent such as in our patient. The lack of imaging abnormalities in patients with minimal or mild encephalopathic symptoms may account for why AE may be underdiagnosed in the acute phase [1,3,7,8,9]. In our case, bilateral blepharospasm, polyminimyoclonus and dystonic posturing of the left hand were novel features which may be fruitful for further research [1,2,3,4,5,6,7,8,9,10,11,12,13,14,15,16,17,18,19,20,21,22,23,24,25].

There is growing clinical, epidemiological and experimental evidence that support the hypothesis of aluminium-induced encephalomyelopathy [1,2,3,4,5,6,7,8,9,10,11,12,13,14,15,16,17,18,19]. In our case, the presentation at age 45 years was in temporal relation with an acute exposure to mixed paints containing 30% of aluminium plus the progression of the disease with chronic exposure to the putative toxin have established a positive temporal relationship. We have described supportive evidence in the literature of the plausibility of the cause effect hypothesis on aluminium toxicity. We also found a positive biological gradient because the disease progressed with chronic exposure without the use of personal protective equipment. Exponential reduction of serum aluminium levels with progressive increment of MMSE score (***Figure 2***) and persistent improvement of abnormal movement disorders (***Video 1 segment 3***) after treatment suggest causal association and therapeutic success [1,2,3,4,5,6,7,8,9,10,11,12,13,14,15,16,17,18,19, 20]. However, the lack of a repeat MRI scan remains a limitation of the study and this is carded for the future.

Only a few cases of aluminum-related encephalopathy or leukoencephalopathy have been reported (***Table 3***). Two of these reported cases featured leukodystrophy without evidence of aluminum exposure [2, 21], but four of those cases could have involved aluminum intoxication [20,22,23,24]. Our case had compelling evidence of exposure to aluminium with hyperaluminemia.

**Table 3:** Summary of the Literature on Aluminum-Related Encephalopathy.


CASE	AGE (YEARS)	GENDER	PROBABLE CAUSE	CLINICAL MANIFESTATIONS	AL CONCENTRATION	PATHOLOGY OR MRI FINDINGS	REFERENCE

1	n.d.	n.d.	Al-containing vaccine	Motor symptoms, cerebellar signs, visual loss, sensory disturbances, cognitive and behavioral problems.	n.d.	MS-like features by MRI	1

2	58	female	Al-containing water	Mental deterioration, visual hallucinations 15 years later.	23.3 µg/g (Reference value <2) in cortex	Congophilic angiopathy	2

3	37	male	Unknown	Mental deterioration for 10 years.	n.d.	Patchy demyelination, calcifications with Al deposits	3

4	52	female	Al-containing cement bone when reconstruction	Loss of consciousness, myoclonic jerks, seizures for 6 months.	9.3 µg/g (Reference value <2) in cortex	Al-containing argyrophilic inclusions in neurons, glia and choroid plexus	4

5	59	female	3.0 g hydroxyl-Al gel for 15 years	Tremor, delirium for 9 months.	195 µg/L (Reference value <10) in serum, 12 µg/L (Reference value <5) in CSF	Nonspecific mild atrophy, calcification	5

6	20	female	Unknown	Mental deterioration, epilepsy.	n.m.	Cystic and spongy white matter, Al deposits in the myelin sheath	6

Our patient	55	female	30% Al-containing marine grade paints for 12 years	Intention tremor of the hands and jerks of the upper limbs, polyminimyoclonus and slight dystonic posturing of the left hand, tremor of the head, bilateral blepharospasm and impaired finger tapping, dysdiadochokinesia and dysmetria with spastic ataxic gait and ample sustentation base. Romberg sign positive, dementia, and microcytic anemia. Movement disorders markedly improved or resolved after chelation therapy (Video 1).	Pretherapy: 1,391 µg/L (Reference value 0-9) in serum. Posttherapy: 879 µg/L in serum. Follow up 5 years: 212 in serum.	Brain MRI showed mild cerebral and cerebellar atrophy, with sparing of the corpus callosum, brainstem and cervical-medullary junction.	


**Abbreviations:** n.d., not described; Al, aluminium; MS, multiple sclerosis; CSF, cerebrospinal fluid; n.m., not measured.

In conclusion, we alert clinicians to the possibility that chronic exposure to paints vapors containing high concentration of aluminium can cause toxic encephalopathy with an array of movement disorders encompassing intention tremor of the hands, polyminimyoclonus and dystonic posturing of the hand, tremor of the head, bilateral blepharospasm, impaired finger tapping, dysdiadochokinesia and dysmetria with spastic ataxic gait and ample sustentation base, and dementia. Careful analysis of differentials by criteria for diagnosis, natural history of progression, movement disorders phenomenology, medical assessments including MMSE, aluminium quantification along with the imaging were most useful for this study. To the best of our knowledge, this is the first reported case of reversible movement disorders and dementia associated with hyperaluminemia caused by chronic exposure to marine grade paints containing high grade aluminium [1,2,3,4,5,6,7,8,9,10,11,12,13,14,15,16,17,18,19,20,21,22,23,24,25]. This case also extends the clinical spectrum of hyperaluminemia in man and highlights movement disorders associated with aluminium neurotoxicity.

## ADDITIONAL FILES

The additional files for this article can be found as follows:

10.5334/tohm.588.s1 Table 1. Medical Investigations.

10.5334/tohm.588.s2 Table 2. Causes of Encephalomyelopathy.

## References

[1] Ganrot PO. Metabolism and Possible Health Effects of Aluminum. Environmental Health Perspectives. 1986; 65: 363–441. DOI: 10.1289/ehp.8665363 2940082 PMC1474689

[2] Itoh M, Suzuki Y, Sugai K, et al. Progressive Leukoencephalopathy Associated with Aluminium Deposits in Myelin Sheath. Journal of Child Neurology. 2008; 23: 938–943. DOI: 10.1177/0883073808315413 18660477

[3] Bugiani O, Ghetti B. Progressing encephalomyelopathy with muscular atrophy, induced by aluminium powder. Neurobiol Aging. 1982; 3: 209–222. DOI: 10.1016/j.jtemb.2016.12.001 7162550

[4] Yasui M, Yase Y, Ota K, et al. High aluminum deposition in the central nervous system of patients with amyotrophic lateral sclerosis from the Kii Peninsula, Japan: two case reports. Neurotoxicology. 1991; 12: 277–283. 1956586

[5] Rao JK, Katsetos CD, Herman MM, et al. Experimental aluminum encephalomyelopathy. Relationship to human neurodegenerative disease. Clin Lab Med. 1998; 18: 687–698, viii. DOI: 10.1016/S0272-2712(18)30144-6 9891607

[6] Lederman RJ, Henry CE. Progressive dialysis encephalopathy. Ann Neurol. 1978; 4: 199–205. DOI: 10.1002/ana.410040302 718132

[7] Filley CM, Kleinschmidt-demasters BK. Toxic leukoencephalopathy. N. Engl. J. Med. 2001; 345: 425–432. DOI: 10.1056/NEJM200108093450606 11496854

[8] Strong MJ, Garruto RM. Chronic aluminum-induced motor neuron degeneration: clinical, neuropathological and molecular biological aspects. Can J Neurol Sci. 1991; 18: 428–431. PMID: 1933693. DOI: 10.1017/S0317167100032601 1933693

[9] Krewski D, Yokel RA, Nieboer E, et al. Human health risk assessment for aluminium, aluminium oxide, and aluminium hydroxide. J Toxicol Environ Health B Crit Rev. 2007; (10 Suppl 1): 1–269. DOI: 10.1080/10937400701597766 18085482 PMC2782734

[10] Longstreth WT, Jr, Rosenstock L, Heyer NJ. Potroom palsy? Neurologic disorder in three aluminum smelter workers. Arch Inter Med. 1985; 145: 1972–5. DOI: 10.1001/archinte.145.11.1972 4062445

[11] Assad N, Sood A, Campen MJ, Zychowski KE. METALS-INDUCED PULMONARY FIBROSIS. Curr Environ Health Rep. 2018; 5: 486–98. DOI: 10.1007/s40572-018-0219-7 30298344 PMC6310083

[12] Ramcharan K, Ramesar A, Ramdath M, Teelucksingh J, Gosein M. Encephalopathy and Neuropathy due to Glue, Paint Thinner, and Gasoline Sniffing in Trinidad and Tobago-MRI Findings. Case Reports in Neurological Medicine. 2014; 2014: Article ID 850109, 4 pages. DOI: 10.1155/2014/850109 PMC4087279 25045557

[13] Kim Y, Kim JW. Toxic Encephalopathy. Saf Health Work. 2012; 3: 243–56. Published online 2012 Nov 30. DOI: 10.5491/SHAW.2012.3.4.243 23251840 PMC3521923

[14] Anghes JR, Schreader MT. EEG in dialysis encephalopathy. Neurology. 1980; 30: 148–54. DOI: 10.1212/WNL.30.11.1148 7191512

[15] Valk J, van der Knaap S. Toxic Encephalopathy. AJNR. 1992; 13: 747–60. 0195-6108/92/1302-0747. 1348902 PMC8333221

[16] Jung KH, Chu K, Kim YA, Jeon BS. Rapidly Progressive Toxic Leukoencephalomyelopathy with Myelodysplastic Syndrome: a Clinicopathological Correlation. J Clin Neurol. 2007; 3: 45–9. Published online 2007 Mar 20. DOI: 10.3988/jcn.2007.3.1.45 19513342 PMC2686928

[17] Filley CM, Heaton RK, Rosenberg NL. White matter dementia in chronic toluene abuse. Neurology. 1990; 40: 532–34. DOI: 10.1212/WNL.40.3_Part_1.532 2314597

[18] Kornfeld M, Moser AB, Moser HW, et al. Solvent vapor abuse leukoencephalopathy. Comparison to adrenoleukodystrophy. J Neuropathol Exp Neurol. 1994; 53: 389–8. DOI: 10.1097/00005072-199407000-00011 8021713

[19] Inan-Eroglu E, Ayaz A. Is aluminum exposure a risk factor for neurological disorder? J Res Med Sci. 2018; 23: 51 Published online 2018 Jun 6. DOI: 10.4103/jrms.JRMS_921_17 30057635 PMC6040147

[20] Exley C, Esiri MM. Severe cerebral congophilic angiopathy coincident with increased brain aluminium in a resident of Camelford, Cornwall, UK. J Neurol Neurosurg Psychiatry. 2006; 77: 877–9. DOI: 10.1136/jnnp.2005.086553 16627535 PMC2117501

[21] Lapresle J, Duckett S, Galle P, Cartier L. Clinical, anatomical and biophysical data on a case of encephalopathy with aluminum deposits. C R Seances Soc Biol Fil. 1975; 169: 282–5. 126756

[22] Authier FJ, Cherin P, Creange A, et al. Central nervous system disease in patients with macrophagic myofasciitis. Brain. 2001; 124: 974–83. DOI: 10.1093/brain/124.5.974 11335699

[23] Reusche E, Pilz P, Oberascher G, et al. Subacute fatal aluminum encephalopathy after reconstructive otoneurosurgery: a case report. Hum Pathol. 2001; 32: 1136–40. DOI: 10.1053/hupa.2001.28251 11679949

[24] Shirabe T, Irie K, Uchida M. Autopsy case of aluminum encephalopathy. Neuropathology. 2002; 22: 206–10. DOI: 10.1046/j.1440-1789.2002.00432.x 12416561

[25] Bondy SC. The neurotoxicity of environmental aluminum is still an issue? Neurotoxicology. 2010; 31: 575–81. Published online 2010 May 27. DOI: 10.1016/j.neuro.2010.05.009 20553758 PMC2946821

