# Supplementary material for: Movement Disorders and Dementia in a Woman With Chronic Aluminium Toxicity: Video-MRI Imaging
Source: Tremor Other Hyperkinet Mov (N Y). 2021 Feb 1;11:5. doi: 10.5334/tohm.588 (PMC7863844; doi:10.5334/tohm.588)
Supplement: Table 1. — Medical Investigations. [file tohm-11-1-588-s1.pdf]

Table 1
Medical Investigations

Blood test	Result	Reference range
White cell count	8.3×10^9/L	4.5–11.0×10^9/L
Hemoglobin	**108 g/L**	**120-160 g/L (women)**
Mean corpuscular volume	**72 fL**	**80–96 fL**
Platelet count	281×10^3/μL	150–400×10^3/μL
Chloride	101 mmol/L	96-106 mmol/L
Potassium	4.1 mmol/L	3.5–5.1 mmol/L
Sodium	138 mmol/L	135–145 mmol/L
Magnesium	0.9 mmol/L	0.65-1.05 mmol/L
Phosphorus	1.1 mmol/L	1.0-1.5 mmol/L
Fasting glucose	4.9 mmol/L	3.9-6.1 mmol/L
Creatinine	86 μmol/L	50-110 μmol/L
Urea	7.2 mmol/L	2.9-8.2 mmol/L
Uric acid	320 μmol/L	120-420 μmol/L
Alanine aminotransferase	32 IU/L	20–60 IU/L
Aspartate aminotransferase	28 IU/L	5–40 IU/L
γ-glutamyl transpeptidase	21 IU/L	8–61 IU/L
Lactate dehydrogenase	85 IU/L	45-90 IU/L
Alkaline phosphatase	103 IU/L	40–160 IU/L
Total bilirubin	12 μmol/L	3-22 μmol/L
Conjugated bilirubin	4 μmol/L	0-5 μmol/L
Protein	72 g/L	60-80 g/L
Albumin	42 g/L	35-55 g/L
Albumin-corrected calcium	9.90 mg/dL	9.6–11.2 mg/dL
International normalized ratio	0.9	0.5–1.1
C-reactive protein	1.0 mg/dL	0.0-1.0 mg/dL
Rheumatoid factor	18 IU/mL	0.0-20 IU/mL
Erythrocyte sedimentation Rate	10 mm/h	0.0-15 mm/h
Total cholesterol	4.5 mmol/L	< 5.2 mmol/L
Triglycerides	1.3 mmol/L	0.45-1.71 mmol/L
Amylase	106 IU/L	25-125 IU/L
Iron	21 μmol/L	5-29 μmol/L (women)
Ferritin	120 μg/L	11-307 μg/L (women)
Transferrin	102 mg/dL	204-350 mg/dL
Transferrin saturation	23%	20%-50%
Creatine phosphokinase	110 IU/L	26-192 IU/L
Toxoplasma gondii IgG antibodies	0.43	0.0-0.54
Toxoplasma gondii IgM antibodies	0.33	0.0-0.54
Herpes virus 1 IgG antibodies	0.50	0.0-0.8
Herpes virus 1 IgM antibodies	0.40	0.0-0.8
Herpes virus 2 IgG antibodies	0.30	0.0-0.8
Herpes virus 2 IgM antibodies	0.50	0.0-0.8
Lead	2.0 μg/dL	0.0-5 μg/dL
Mercury	8.0 μg/L	0.0-20 μg/L
Arsenic	25 μg/L	0.0-49 μg/L
Chromium	1.0 μg/100 mL	2.0-3.0 μg/100 mL
Copper	95 μg/dL	70-150 μg/dL
Ceruloplasmin levels	30 g/mL	22.9–43.1 g/mL
Zinc	96 μg/dL	90-150 μg/dL
Thyroid stimulating hormone	3.8 mIU/L	0.4-4.8 mIU/L
Triiodothyronine	2.0 nmol/L	1.1-2.9 nmol/L
Total Thyroxine	101 nmol/L	66-155 nmol/L
Thyroid peroxidase antibody	15 IU/mL	0.0-34 IU/mL
Thyroglobulin antibody	10 IU/mL	0.0-19 IU/mL
Thyroid-stimulating immunoglobulin antibody	52% of basal activity	0.0-139% of basal activity
Thyroid-stimulating hormone receptor binding inhibitor immunoglobulin	0.85 IU/L	0.0-1.75 IU/L
Vitamin B12	452 pg/mL	160-950 pg/mL
Folic acid	5.7 ng/mL	2.7-17.0 ng/mL
Methylmalonic acid	0.1 μmol/L	0.00-0.40 μmol/L
Anti-double stranded DNA test	12.0 IU/mL	0.0-29.0 IU/mL
Anti-nuclear factor	1.0 IU	Negative: 0.0-1.0 IU<br>Weakly positive: 1.1-2.9 IU<br>Positive: 3.0-5.9 IU<br>Strongly positive: ≥6.0 IU
Anti-nuclear antibody test	0:18	0.0-1:40
Anti-Smith antibodies	0.2 IU/mL	Negative: 0.0-6 IU/mL<br>Equivocal: 7.0-30 IU/mL<br>Positive: ≥31 IU/mL
Perinuclear anti-neutrophil cytoplasmic antibodies	0.8 EU/mL	0.0-10 EU/mL
Anti-neutrophil cytoplasmic antibodies	9 AU/mL	Negative: ≤19 AU/mL<br>Equivocal: 20-25 AU/mL<br>Positive: ≥26 AU/mL
Cancer antigen 15-3	12 IU/mL	0.0-30 IU/mL
Cancer antigen 19-9	15 IU/mL	0.0-37 IU/mL
Cancer antigen 125	13 IU/mL	0.0-35 IU/mL
Cancer antigen 195	1.0 IU/mL	0.0-8.3 IU/ml
Carcinoembryonic antigen	1.8 μg/L	< 3.0 μg/L
Alpha-fetoprotein	12 ng/mL	10-20 ng/mL
Anti-cyclic citrullinated peptides	9 IU/mL	0.0-19 IU/mL
Venereal disease research laboratory (VDRL)	Non-reactive	Non-reactive or reactive

Fluorescent *Treponema pallidum* antibody absorption	Negative	Positive or negative
ELISA for HIV	Non-reactive	Non-reactive or reactive
Mantoux Test	Negative	Positive or negative
QuantiFERON Test	Negative	Positive or negative
HTLV-I and HTLV-II antibodies	Negative	Negative or Positive
Hepatitis B surface antigen	Negative	Positive or negative
Hepatitis C IgG antibodies	Negative	Positive or negative
Hepatitis C IgM antibodies	Negative	Positive or negative
Six panel drug screening in urine: cocaine, amphetamine, methamphetamine, marijuana, opiates, phencyclidine.	Negative	Negative or positive
Anti-Hu antibody, MOG auto-antibodies, NMDAR antibodies, antibodies to aquaporin-4 antigen, immunoglobulin subclass 4, and anti-Glutamic acid decarboxylase antibodies by ELISA.	Tests not obtained	Not Applicable
**Other tests**	**Result**	**Reference range**
Electrocardiogram	Normal	Normal or abnormal
Echocardiogram	Normal	Normal or abnormal
Chest x-ray	Normal	Normal or abnormal
Scalp electroencephalogram	Normal	Normal or abnormal
USS breast and pelvic	Normal	Normal or abnormal
Mammogram	Normal	Normal or abnormal
CT scan of chest, abdomen and pelvic	Normal	Normal or abnormal
EMG/Nerve conduction studies	Normal	Normal or abnormal
MRI scan of brain	**Mild brain and cerebellar atrophy**	**Normal or abnormal**
MRA scan of brain	Normal	Normal or abnormal
MRI scan of spine	Normal	Normal or abnormal
Functional MRI, PET, DTI, MRS and SPECT scans of brain. Sensory evoked potentials.	Not available	Not Applicable
**CSF analysis**	**Result**	**Reference range**
Opening CSF pressure	14 cm of water	6-25 cm of water
Cell count	0 cells/mm^3	0-5 cells/mm^3
Protein	26.3 mg/dL	5-40 mg/dL
Glucose	65 mg/dL	50-80 mg/dL
Culture	No bacterial growth	No bacterial growth or bacterial growth
Cytology	Negative for neoplastic cells	Negative or positive for neoplastic cells
India ink for Cryptococcus neoformans	Negative	Positive or negative
Real-time PCR test for *Escherichia coli*, *Hemophilus influenzae*, *Listeria monocytogenes*, *Neisseria meningitides*, *Streptococcus agalactia*, *Streptococcus pneumonia*, Cytomegalovirus DNA, Enterovirus, Herpes simplex virus 1, Herpes simplex virus 2, Human herpes virus 6, human parechovirus, Varicella zoster virus, Epstein Barr virus, parvovirus, Lymphocytic choriomeningitis virus, Toxoplasma gondii and Cryptococcus neoformans gattii.	Not detected	Detected or not detected
Oligoclonal bands	No bands in CSF detected	Bands detected or not detected
VDRL	Non-reactive	Non-reactive or reactive

CSF, cerebrospinal fluid; ELISA, enzyme-linked immune sorbent assay.
Values written in bold indicate abnormal values.
