# Supplementary material for: Movement Disorders and Dementia in a Woman With Chronic Aluminium Toxicity: Video-MRI Imaging
Source: Tremor Other Hyperkinet Mov (N Y). 2021 Feb 1;11:5. doi: 10.5334/tohm.588 (PMC7863844; doi:10.5334/tohm.588)
Supplement: Table 2. — Causes of Encephalomyelopathy. [file tohm-11-1-588-s2.pdf]

Table 2
Causes of Encephalomyelopathy.

Type	Causes of encephalomyelopathy
**Vascular/Hypoxic**	Hypoxia, ischemia, infarction, thrombosis, low cardiac output, atherosclerosis, myocardial infarction, CNS vasculitis, Wegener's granulomatosis, polyarteritis nodosa, arteriovenous malformation or fistula, choking game, strangulation, ulegyria.
**Autoimmune**	Paraneoplastic and non-paraneoplastic, CNS vasculitis, Sjögren's syndrome, systemic lupus erythematosus, hypereosinophilic syndrome, Behçet's disease, multiple sclerosis, scleroderma, Hashimoto's encephalopathy, stiff person syndrome, Guillain-Barré syndrome, post-infection acute transverse myelitis.
**Mitotic**	Primary and secondary brain or spinal cord tumors, liver cancer, intravascular B cell lymphoma, paraneoplastic myelopathy.
**Drug-induced**	Tacrolimus, cyclosporine, mycophenolate, azathioprine, rituximab, amiodarone, carbamazepine, indomethacin, methotrexate, etoposide, methapyrilene, halothane, nitrous oxide.
**Infective**	**Viral:** HIV seroconversion, AIDS-related myelopathy, HTLV-I or II, cytomegalovirus, Epstein-Barr, herpes simplex, varicella-zoster, adenovirus, enterovirus, coxsackie B, herpes 6, West Nile, enteroviruses, rabies, JC polyomavirus, and hepatitis B and C. **Bacterial:** Treponema pallidum, Mycobacterium tuberculosis, Neisseria meningitides, Salmonella sp, Shigella sp, Borrelia sp, group B streptococcus. **Fungal:** Cryptococcus neoformans, aspergillus sp. **Parasite:** Toxoplasma gondii.
**Uremic**	End Stage Kidney Disease, Acute Kidney Injury, Renal osteodystrophy causing spondylotic myelopathy.
**Nutritional Metabolic**	Vitamin B1 deficiency, Wernicke encephalopathy, Hypercalcemia, hypocalcemia, hyponatremia, hypernatremia, hypoglycemia, hyperglycemia, copper deficiency, vitamin E deficiency, Lathyrism, Konzo.
**Hypertensive**	Arterial hypertension essential and or organic.
**Physical agents**	Trauma, altitude sickness, outer space exposure, hypothermia, electrical injury, decompression sickness, ionizing radiation.
**Toxic**	Lead, mercury, aluminum, zinc, ammonia, arsenic, cyanide, carbon monoxide, toluene, xylene, benzene, allyl alcohol, ethanol, heroin, formaldehyde, acetates, α-amanitin, aflatoxin, carbon tetrachloride, aroclor 1254, luteoskyrin, monocrotaline, pyrrolizidine alkaloids, diquatdibromide, chlorotoxin, dimethylformamide, diethylnitrosamine, curare, 3-methylcholanthrene, polyethylene glycol, manganese, tetrodotoxin, tetanus toxin, botulinum toxin, conotoxin, tetraethylammonium, bungatoxin, 251-NBOMe, JWH-018, dopamine, glutamate, and nitric oxide.
**Genetic**	Mitochondrial encephalopathy, glycine encephalopathy, X-linked adrenoleukodystrophy, Fredrich's ataxia, hexosaminidase deficiency, Leber's hereditary optic neuropathy, Arnold-Chiari malformation, hereditary spastic paraplegia, neurodegeneration with brain iron accumulation, metachromatic/orthochromatic leukodystrophy, Machado-Joseph disease, abetalipoproteinemia.
**Other causes**	Multiple system atrophy, Parkinson's disease, Alzheimer's disease, hydrocephalus, neurosarcoidosis, lymphomas, brain tumors, brain metastatic cancer, increased intracranial pressure, glycine encephalopathy, mitochondrial encephalopathy, static encephalopathy, progressive multifocal leukoencephalopathy, depression pseudo dementia, other subcortical dementia forms, encephalomyopathies, amyotrophic lateral sclerosis, sarcoidosis, herniated disc, spinal cord necrosis, spinal cord compression, syringomyelia, primary lateral sclerosis, disseminated sclerosis, dopa-responsive dystonia, stiff person syndrome, cervical spondylotic myelopathy, fibromyalgia, chronic subdural hematoma, neuromyelitis optica, arachnoiditis ossificans, tethered cord syndrome, Prion proteins.
